# Impact of an intensive facility-community case management intervention on 6-month HIV outcomes among select key and priority populations in Uganda

**DOI:** 10.1186/s12981-022-00486-9

**Published:** 2022-12-05

**Authors:** David B. Meya, Agnes N. Kiragga, Elizabeth Nalintya, Grace Banturaki, Joan Akullo, Phillip Kalyesubula, Patrick Sessazi, Hillary Bitakalamire, Joseph Kabanda, Julius N. Kalamya, Alice Namale, Moses Bateganya, Joseph Kagaayi, Steve Gutreuter, Michelle R. Adler, Kiren Mitruka

**Affiliations:** 1 Infectious Diseases Institute, College of Health Sciences, Makerere University, Mulago Hill Road, 22418 Kampala, Uganda; 2 Department of Medicine and International Health, University of Minnesota, Minneapolis, MN USA; 3 School of Medicine, College of Health Sciences, Makerere University, Kampala, Uganda; 4 U.S Centers for Disease Control and Prevention, Kampala, Uganda; 5 Rakai Health Sciences, Program, Rakai Uganda; 6 U.S Centers for Disease Control and Prevention, Atlanta, GA USA; 7 Kalangala District Health Services, Kalangala, Uganda; 8 Makerere School of Public Health, College of Health Sciences, Makerere University, Kampala, Uganda

**Keywords:** Antiretroviral therapy, Retention in care, Key populations, Viral suppression, HIV

## Abstract

**Introduction:**

Key and priority populations (with risk behaviours and health inequities) are disproportionately affected by HIV in Uganda. We evaluated the impact of an intensive case management intervention on HIV treatment outcomes in Kalangala District, predominantly inhabited by fisher folk and female sex workers.

**Methods:**

This quasi-experimental pre-post intervention evaluation included antiretroviral therapy naïve adults aged ≥ 18 years from six health facilities in the pre-intervention (Jan 1, 2017–December 31, 2017) and intervention phase (June 13, 2018–June 30, 2019). The primary outcomes were 6-month retention and viral suppression (VS) before and after implementation of the intervention involving facility and community case managers who supported participants through at least the first three months of ART. We used descriptive statistics to compared the characteristics, overall outcomes (i.e., retention, lost to follow up, died), and VS of participants by phase, and used mixed-effects logistic regression models to determine factors associated with 6-month retention in care. Marginal (averaging over facilities) probabilities of retention were computed from the final multivariable model.

**Results:**

We enrolled 606 and 405 participants in the pre-intervention and intervention phases respectively. Approximately 75% of participants were aged 25–44 years, with similar age and gender distributions among phases. Approximately 46% of participants in the intervention were fisher folk and 9% were female sex workers. The adjusted probability of 6-month retention was higher in the intervention phase, 0.83 (95% CI: 0.77–0.90) versus pre-intervention phase, 0.73 (95% CI: 0.69–0.77, p = 0.03). The retention probability increased from 0.59 (0.49–0.68) to 0.73 (0.59–0.86), p = 0.03 among participants aged 18–24 years, and from 0.75 (0.71–0.78) to 0.85 (0.78–0.91), p = 0.03 among participants aged ≥ 25 years. VS (< 1,000 copies/mL) was approximately 87% in both phases.

**Conclusions:**

After implementation of the case management intervention, we observed significant improvement in 6-month retention in all age groups of a highly mobile population of predominantly fisher folk.

## Background

Among 37.7 million people living with HIV worldwide, an estimated 20.7 million lived in eastern and southern Africa at the end of 2020, highlighting the disproportionate HIV burden in the region [1]. Key populations (KP) (e.g., sex workers, men who have sex with men, people who inject drugs, transgender people, people in prisons) and their sexual partners accounted for 65% of 1.5 million new HIV infections globally and 28% of 730,000 new infections in eastern and southern Africa [1]. A key driver of HIV transmission is virologic non-suppression, which can occur among persons unaware of their HIV status, not linked to antiretroviral therapy (ART), and failing ART because of poor adherence or retention [2]. Linkage and retention are particularly challenging among KP and priority populations (PP) (e.g., fisher folk, truckers, uniformed forces, migrant workers, adolescent girls and young women) [3, 4]. These subgroups are not only at high risk of acquiring HIV infection due to risky behavior, but once infected, they are less likely to seek HIV care because of provider and self-stigma, high mobility, misinformation about treatment, and lack of resources [5].

Although the prevalence of HIV among 15–49 year-olds in Uganda is 6.2%, the prevalence among key populations is substantially higher at 13–31% [6]. Among priority populations, fishing communities are disproportionately affected by HIV compared with other trading and agrarian communities [4, 7–10]. Further, fisher folk tend to have low ART coverage, with fewer than half of newly diagnosed persons linked to ART [4, 11, 12], which increases their risk for viral non-suppression and ongoing HIV transmission. Thus, strategies customized for key and priority populations are urgently needed to control the HIV epidemic.

Case management has been shown to improve early retention in care among recently diagnosed HIV-infected adults and improve viral suppression (VS) among enrolees of drug and alcohol addiction treatment programs [13–19]. However, case management studies conducted in Africa have shown mixed results in improving HIV outcomes. For instance, while improved 24-month retention and early ART initiation was demonstrated through case management interventions in Sierra Leone [20], Eswatini [21], Tanzania [22] and Malawi [23], retention was not improved via mobile phone-based case management among pregnant women in South Africa [24].

The Uganda Ministry of Health guidelines for HIV prevention, care, and treatment suggest interventions aimed at reducing HIV incidence among key and priority populations. However, case management has not been formally recommended [25]. To better understand the effectiveness of case management in improving HIV outcomes among KP and PP in Uganda, we implemented and evaluated an intensive facility-community case management intervention in Kalangala district, where 64 of its 84 islands are inhabited, predominantly by fishing and farming communities. Female sex workers compose a relatively high proportion of these communities compared with other districts in Uganda. With an adult (aged > 15 years) HIV prevalence of 18% [26], approximately 15,000 HIV-infected individuals residing on the islands have poor access to HIV care and treatment [27]. The district has 15 health facilities on 12 islands, requiring a boat for transportation.

The aim of the facility-community case management intervention was to provide intensive support to persons newly diagnosed with HIV during at least the first three months of ART.

## Methods

### Study design and participants

We used a pre- and post-intervention study design to evaluate 6-month retention and VS before and after implementation of an intensive case management intervention in six health facilities in Kalangala District, Uganda, supported by the Centers for Disease Control and Prevention (CDC) under the President’s Emergency Plan for AIDS Relief. Three facilities were < 2 h and three were > 2 h from the mainland by boat. Data from the first medical visit following an HIV-positive test onwards were abstracted from medical records, allowing for 6-month follow-up.

For both the pre-intervention and intervention phases, we consecutively enrolled eligible participants, which included ART-naïve adults aged ≥ 18 years who had initiated ART as part of HIV care at one of the six health facilities at least once, following an HIV positive test result. For the intervention phase, only persons who consented to receive the intervention had their data abstracted. The six facilities comprised the primary sampling units. Assuming a 40% relative improvement in retention (from approximately 50% to 90%) in the intervention phase, with an inter-cluster correlation coefficient of 0.05, we calculated a minimum sample size of 340 participants in each phase, with 80% power to detect the assumed difference in retention between the phases.

Ethical approval was granted by the Joint Clinical Research Center in Uganda and the protocol was registered with the Uganda National Council of Science & Technology. This project was reviewed in accordance with CDC human research protection procedures and was determined to be research, but CDC investigators did not interact with human subjects or have access to identifiable data or specimens for research purposes.

### Standard of HIV care

During the pre-intervention phase, the standard of HIV care included the performance of a baseline CD4 test, monthly clinical follow-up for the first three months on ART then every 2–3 months for ART refills, screening for opportunistic infections, cotrimoxazole prophylaxis, and viral load (VL) testing conducted at 12 months after ART initiation, thereafter annually if virally suppressed. Health care workers from the health facilities conducted visits monthly at facility-managed outreach sites in the community (e.g., boat landing sites) to provide HIV testing, counselling, and ART initiation and refills. The case manager cadre did not exist, instead facility staff encouraged newly diagnosed individuals to return for follow-up care at the facility or their outreach sites. Health facilities utilized village health teams (VHT) to track persons who did not return for follow-up. Tracking was conducted via home visits and phone calls.

During the intervention phase, the World Health Organization’s *Treat All* policy (implemented in Kalangala in December 2017) was adopted, making all people with HIV eligible for ART [28]. Virologic testing was implemented at six and twelve months after ART initiation, thereafter annually if virally suppressed. To improve linkage and retention at ART facilities nationwide, linkage facilitators and volunteer counsellors were employed to support outreach activities and tracking of missed appointments.

### Case management intervention

The implementation of the intervention was initiated in May 2018 with recruitment and training of community and facility case managers. Each of the six health facilities was assigned 2–3 facility case managers (FCM) and 1–2 community case managers (CCM), for a total 15 FCM and 17 CCM. During the intervention phase, newly diagnosed HIV-infected persons initiating ART were provided written consent to be followed by any one of the 3–5 FCM and CCM assigned to the community for the first 3 to 6 months after initiating ART.

FCM were expert clients familiar with the facilities, where they supported medical chart retrieval and triaging. Each FCM was expected to follow 4–6 participants. FCM oriented participants to HIV care, ensured that standard of care was provided, and rendered ongoing supportive counseling to manage barriers to adherence, including stigma and discrimination.

Community case managers, recruited from the VHT serving within 10 km of each facility, signed a confidentiality agreement and received training on HIV adherence counseling and case management, mobilized and educated the community on health issues. CCM followed 10–15 participants and ensured that those diagnosed at outreach sites registered at the facility, assessed barriers to care, tracked missed appointments via phone call, text, or home visits, and delivered ART refills to those unable to attend the clinics. They coordinated tracing with VHTs when participants relocated and liaised with community support groups to mobilize the community in HIV prevention and treatment. CCM also ensured participants received pre-appointment reminders and encouraged participants’ household members to test for HIV and distributed vouchers to be presented at the facilities or at outreach sites to facilitate fast tracking through the clinic. CCM interacted with participants at least two times per month and documented the status of the participant (e.g., alive on ART, LFU, died), adherence, and the presence of any symptoms in their diaries. CCM and FCM for each facility met once per month to review missed appointments and collaborated with peer counsellors and linkage facilitators to ensure treatment continuity.

### Data collection

In both phases an abstraction form was used to collect de-identified data from electronic and paper records documenting their medical visits for HIV care services. Data included demographics, care-entry point (main health facilities or their outreach locations), HIV diagnosis date, ART initiation date, WHO HIV stage, CD4, HIV VL test date and result, and monthly care status (i.e., transferred, died, alive and retained care, and LTFU). During the intervention phase, case managers’ diaries were also used as a data source to assess participants’ care status. Study staff reviewed the diaries for completeness of entries weekly, including details of how case managers contacted participants, whether participants had relocated, and if a future visit was scheduled. Further, facility nurses-in-charge were asked to verify case manager interactions with participants when they presented for ART refill. Data from the abstraction forms were entered into a REDCap database, which was assessed for data quality and completeness through regular queries.

### Statistical analysis

We excluded participants who transferred out of the six facilities from the analysis. The main outcomes assessed were 6-month retention and VS. Based on the President’s Emergency Plan for AIDS Relief (PEPFAR) Monitoring, Evaluation, and Reporting indicators (version 2.2), retention was defined as having a documented provider visit, ART pick-up at the facility, or encounter with a case manager in the community, within 90 days after the scheduled appointment date [29]. VS was defined as having < 1,000 copies/ml. Available VL results within 90 days prior to or after the scheduled follow-up visit were included in the analysis.

Among the remaining participants meeting the inclusion criteria, we used descriptive statistics to compare demographic and clinical characteristics, and 6-month VS (overall and among participants retained) in pre-intervention and intervention phases. We compared frequencies of overall outcomes (i.e., retention, LTFU, and died) by phase.

Logistic regression was conducted to assess factors associated with 6-month retention and to estimate retention probabilities. The covariates of interest were age range (18–24, 25–34, 35–44, and 45 and older), sex, WHO stage (I–IV), entry point (facilities or their outreach sites) and phase (pre-intervention and intervention). Among the 1,011 participants who did not transfer out during the study, there were 1, 51, 21, and 55 missing observations for sex, WHO stage, age range, and entry point, respectively. These missed observations were completed in ten datasets using multiple imputation chained equations [30]. Mixed-effects logistic regression models including an intercept-level random effects for site were used for inference to determine associations with retention in care. Models were fitted for each of the ten imputed datasets, and results were combined using Rubin’s rules [31]. We began with a full model including sex, age group, WHO stage, entry point, phase, and four two-way interaction terms (i.e., sex × phase, entry point × phase, age × phase, and sex × age). We sequentially deleted insignificant interaction terms, followed by insignificant covariates and levels of categorical covariates that did not meet a threshold of *P* ≤ 0.05 based on *t* or *F* tests for binary or categorical covariates, respectively. The marginal (averaging over sites) estimates of retention were obtained from the final multivariable model fitted to the multiply imputed data. All statistical analyses were performed using STATA 16.1, (StataCorp, College Station, TX).

## Results

### Characteristics of participants

Of the 641 HIV-infected persons screened in the pre-intervention phase, 35 were excluded (aged < 18 years, n = 20; missing data, n = 9; transferred out, n = 6)). Of the 497 persons screened in the intervention phase, 92 were excluded (currently on ART, n = 76; aged < 18 years, n = 4; transferred out, n = 12).

Among 1,011 participants included in the analysis, pre-intervention phase (n = 606) and intervention phase (n = 405) participants were generally similar in age (with almost half aged 25–34 years), gender, and WHO stages, with few participants in WHO stages III and IV (Table 1). The facility outreach sites enrolled 85.2% of the participants in the intervention phase, but only 24.8% in the pre-intervention phase.

**Table 1:** Characteristics of antiretroviral therapy naïve, HIV-infected persons by evaluation phase of case management intervention, Kalangala District, Uganda

Characteristic	Pre-intervention phase N = 606 (%)	Intervention phase N = 405 (%)	Total 1,011 (%)
Age (years)			
18–24	79 (13.0)	60 (14.8)	139 (13.8)
25–34	292 (48.2)	201 (49.6)	493 (48.8)
35–44	169 (27.9)	112 (27.7)	281 (27.8)
> 44	45 (7.4)	32 (7.9)	77 (7.6)
Missing data	21 (3.5)	0 (0.0)	21 (2.1)
Sex			
Female	326 (53.8)	202 (49.9)	528 (52.2)
Male	280 (46.2)	202 (49.9)	482 (47.7)
Missing data	0 (0)	1 (0.3)	1 (0.1)
WHO stage			
I	397 (65.5)	271 (66.9)	668 (66.1)
II	164 (27.1)	77 (19.0)	241 (23.8)
III	42 (6.9)	6 (1.5)	48 (4.7)
IV	1 (0.2)	2 (0.5)	3 (0.3)
Missing Data	2 (0.3)	49 (12.0)	51 (5.0)
Entry point			
Facility outreach	150 (24.8)	345 (85.2)	495 (49.0)
Main facility	401 (66.2)	60 (14.8)	461 (45.6)
Missing data	55 (9.1)	0 (0.0)	55 (5.4)

Among 405 participants in the intervention phase, 40.5% were fishermen, 14.3% were housewives, 9.9% were farmers, 9.1% were female sex workers, 7.4% were palm oil plantation workers, 5.4% were trading in fish, and 13.3% were involved in various commercial activities. Data on occupation were not available for the pre-intervention phase.

### Overall outcomes

Table 2 shows the overall outcomes of 1,011 participants. During the pre-intervention phase, fewer participants (72.6%) were retained in care after six months compared with the intervention phase (82.8%). The pre-intervention phase had a higher proportion of participants who were LTFU and died, and the intervention phase had a higher proportion of participants who transferred out.

**Table 2:** Six-month outcomes among antiretroviral therapy naive HIV-infected persons by evaluation phase of a case management intervention, Kalangala District, Uganda

Overall outcomes	Pre-intervention phase N = 606	Intervention phase N = 405	Total N = 1,011
	n (%)	n (%)	n (%)
Retained at 6 months	440 (72.6)	336 (82.8)	776 (76.8)
Lost to follow-up	154 (25.4)	64 (15.8)	218 (21.6)
Died	12 (2.0)	5 (1.2)	17 (1.7)

18 participants who transferred out excluded: 6 pre-intervention phase and 12 intervention phase

### Implementation of intervention

During the intervention phase, CCM interacted with the participants for a median of 5 months (IQR: 3, 6). Among the 405 participants in the intervention phase, a total of 196 (48.4%) participants interacted with the CCM during home visits alone, 183 (45.2%) by phone alone, 18 (4.4%) through text messaging only, 4 (1.0%) via both phone calls and home visits, 3 (0.74%) using both text messaging and home visits, and 1 (0.25%) via text messaging and phone calls. Of the total 2307 encounters/interactions documented by the 32 facility and community case managers, 58% occurred during the first three months after ART initiation and 42% during 4–6 months of ART initiation. There was no difference in the number of case manager interactions between participants who initiated ART at the facility [mean (SD) 4.7 (1.9) versus at an outreach site, mean (SD) 4.5(1.9)]. Of 189 participants who had missed a monthly scheduled health facility appointment during the intervention phase, the case-managers successfully re-engaged 77 (41%) participants into medical care through home visits, phone calls or text messaging. Of those 77 participants, 7 (9.1%) had missed a scheduled appointment by 0–28 days, 43 (55.8%) by 29–60 days, 11 (14.3%) by 61–90 days, and 16 (20.8%) by more than 90 days.

Of the 57 testing vouchers distributed to participants in the intervention phase, 28 (49%) were returned by household members who presented for an HIV test, and of those, 9 (32%) tested HIV positive.

### Viral load testing and suppression

VL test results were available for 502 (49.7%) of 1,011 participants (48.7% [295/606] pre-intervention and 51.1% [207/405] intervention phase). Of those participants with results, approximately 87% had VS in each phase (259/295 pre-intervention and 181/207 intervention). Among participants retained at six months, a slightly higher proportion had available VL results in the pre-intervention phase, 63.9% (281/440) versus 58.9% (198/336) in the intervention phase; of those, the proportion with VS was approximately 87% in each phase (249/281 pre-intervention and 173/198 intervention). The median duration from ART initiation to VL testing was 10 months and 8 months for pre-intervention and intervention phase, respectively.

### Factors associated with 6-month retention

None of the two-way interaction terms, including sex and phase, were significantly associated with retention. Among the covariates, only phase and age were significantly associated with retention at six months (Table 3). The odds ratios for the effect of age groups 35–44 years and 45 years and older were indistinguishable from 1.0, therefore those levels were combined with the referent age group 25–34 years in the adjusted analysis. Averaging over sites and participants, the estimated probability of retention was statistically significantly higher in the intervention phase (0.83 [CI 0.77–0.90]) versus pre-intervention phase (0.73 [CI 0.69–0.77], p = 0.03 and higher among participants in both age groups (18–24 years and ≥ 25 years) in the intervention versus pre-intervention phase (p = 0.03) (Table 4). Averaging over sites and phases, the estimated probability of retention for participants aged 18–24 years was statistically significantly lower (P < 0.01) compared with that for participants aged ≥ 25 years (0.65 [CI 0.55–0.74] versus 0.79 [CI 0.77–0.81].

**Table 3:** Factors associated with 6-month retention in care among antiretroviral therapy naïve, HIV-infected persons, Kalangala District, Uganda

	Numbers of participants^1^		Crude odds ratios^2^		Adjusted odds ratios^2^	
	Not retained N = 235 N (%)	Retained N = 776 N (%)	Estimate (95% CI)	P-value < 0.01	Estimate (95% CI)	P-value < 0.01
Age						
18–24	49 (21.3)	90 (11.8)	0.50 (0.36–0.70)		0.48 (0.34–0.68)	
25–34	105 (45.7)	388 (51.1)	reference		Combined reference^3^	
35–44	57 (24.8)	224 (29.5)	1.06 (0.91–1.24)			
45 +	19 (8.3)	58 (7.6)	0.82 (0.46–1.44)			
Sex				0.22		
Female	112 (47.7)	416 (53.7)	reference			
Male	123 (52.3)	359 (46.3)	0.79 (0.53–1.16)			
WHO stage				0.33		
I	144 (70.6)	524 (69.3)	reference			
II	42 (20.6)	199 (26.3)	1.30 (0.79–2.18)			
III–IV	18 (8.8)	33 (4.4)	0.56 (0.27–1.17)			
Entry point				0.32		
Facility	118 (53.4)	343 (46.7)	reference			
Outreach	103 (46.6)	392 (53.3)	1.30 (0.78–2.16)			
Phase				0.06		0.05
Pre-intervention	166 (70.6)	440 (56.7)	reference			
Intervention	69 (29.4)	336 (43.3)	1.84 (0.97–3.49)		1.88 (1.00–3.54)	

^1^Column frequencies in the table body are based on the complete-case records prior to imputation

^2^Analyses are based on ten imputations yielded 1,011 completed-case records

^3^Ages 25 and older were recombined into a single reference category

**Table 4:** Retention probabilities^1^ (95% CI) at six months from ART initiation among antiretroviral therapy naïve, HIV-infected persons based on the final multivariable model, Kalangala District, Uganda

	Pre intervention	Intervention	P-value^2^
Phase	0.73 (0.69- 0.77)	0.83 (0.77–0.90)	0.03
Age (years)			
18–24	0.59 (0.49–0.68)	0.73 (0.59–0.86)	0.03
25 and older	0.75 (0.71–0.78)	0.85 (0.78–0.91)	0.03

Age	Age group		
	18–24	25 and older	
	0.65 (0.55–0.74)	0.79 (0.77–0.81)	< 0.01

^1^These are the marginal retention probabilities averaging over the random effect of facilities from the final multivariable model.

^2^Student t tests of differences between columns

## Discussion

In this pre-post study, we compared six-month retention in care before and after implementing a facility-community case management intervention for the first 3–6 months after ART initiation among a highly mobile key and priority population, predominantly fisher folk but also female sex workers, newly diagnosed with HIV in Kalangala District, Uganda. Similar to previous studies in sub-saharan Africa [21–24], our study demonstrated the utility of case management in improving retention in HIV care. Following the implementation of intervention, we observed a ten-percentage point (73% to 83%) improvement in 6-month retention in HIV care. The intervention appeared to be effective in all age groups of a population, that would otherwise be at-risk for attrition, supporting the role of case management to improve retention among KP and PP with HIV.

The case management intervention involved close collaboration between facility and community case managers to understand and address challenges to retention in care and facilitate rapid identification and tracking of clients who missed appointments. A similar strategy involving facility-community-based peer support models was successful in improving retention in Option B + prevention of maternal-to-child HIV transmission in Malawi [23]. In our study, one out of five participants re-engaged in care would have been LTFU (missed appointment by > 90 days). Notably, 75% of 77 participants re-engaged in care had missed a scheduled appointment by > 29 days but < 60 days, demonstrating the potential role of the intervention in improving early retention; poor retention in early care is associated with an increased risk for mortality [32].

During the intervention phase, we observed that after delivery of care at outreach sites, facility staff did not always update facility-based medical records timeously. Case managers’ diaries were an additional data source that allowed us to better document retention. Without the use of case manager’s diaries, 6-month retention in this evaluation would have dropped from 72.7% (pre-intervention) to 66.9% (intervention). Notably, among the Kalangala District facilities that did not participate in the intervention, 6-month retention was estimated to decline from a mean of 74% to 47% during the study period (unpublished program data). Thus, the development of real-time data capture through universal open-source point-of-care (POC) electronic medical record (EMR) systems [33] could be a solution to improving documentation of retention in care among highly mobile populations.

We identified that young adults aged 18–24 years were less likely to be retained in care compared with older participants, independent of other covariates. However, given that the intervention was associated with a substantial improvement in retention among young adults (from 59 to 73%), the lessons learned from the intervention could be adapted to other settings. Young people living with HIV are known to have low rates of retention, adherence, and VS, and higher HIV-related mortality —urgently requiring evidence-based interventions specific to this sub-population [34, 35].

After, adjusting for intervention phase and other variables, male sex was not significantly associated with retention. Studies show men have a higher risk of attrition and less likely to re-engage in care compared with women [36, 37]However, in this highly mobile population of pre-dominantly fisher folk and female sex workers we found no significant difference in 6-month retention between males and females.

Based on previous studies [13, 16], we expected to find a higher proportion of participants in the intervention phase versus pre-intervention phase to have VS. However, in both phases approximately half of the participants had available VL tests, and of those, similar proportions (~ 87%) has VS, both overall and among participants retained at 6 months. These findings represent a key opportunity for further improvement of the case management intervention to ensure that case managers facilitate the conduct and documentation of VL tests and improve adherence. Some ways to make these improvements include providing greater mentorship to case managers around treatment literacy, emphasizing the importance of VL testing, and messaging “undetectable equals untransmissible (U = U)”[38], to promote adherence. In addition better access to VL testing through increased use of dried blood spot (DBS) samples and POC platforms are necessary to improve VL coverage, especially in highly mobile key populations. POC VL testing, shown to be feasible in sub-Saharan Africa [39], and currently being implemented for early infant HIV diagnosis (EID) in Uganda[40].

Similar to Steiner et al. in Tanzania [22], where they sought to determine the prevalence of undiagnosed HIV and the effect of case management linkage interventions in the community, we evaluated the utility of vouchers provided to household contacts to encourage HIV testing. Although only half of the household members who received vouchers presented for testing, and of those, 30% were HIV positive, suggesting that the use of vouchers is potentially a high yield strategy to identify HIV cases in highly mobile populations. However, case managers should emphasize the benefits of HIV of testing to improve the number of contacts who present for testing.

This study had several limitations. First, the case manager diaries could have documented participant interactions that did not occur to demonstrate worth for financial compensation. However, the documentation of interactions with participants were carefully monitored by study staff and verified by the facility nurse-in charge with participants. Second, we could not verify whether persons retained in care were on ART since we did not collect pharmacy data. Third, due to the limited number of participants with a VL result, we were unable to use VS as an objective measure to verify retention on treatment. Fourth, the participants deemed LTFU could have transferred to another facility, which we were unable to verify. Fifth, data quality and completeness were likely better in the intervention period (prospective) versus pre-intervention (retrospective). Finally, we cannot establish direct causality between the intervention and improved 6-month retention. However, improved 6-month retention in the Kalangala District facilities implementing case management versus other facilities in the same district [41] was observed. Further, this pre-post study where case managers were also members of the HIV community, adds to the limited literature about the effectiveness of case management in key and priority populations in resource-limited settings.

## Conclusions

In conclusion, the facility and community case management intervention demonstrated an improvement in 6-month retention compared with the standard of care among highly mobile KP and PP, predominantly fisher folk, but also included female sex workers. The improved retention was observed in all age groups, including persons aged 18–24 years, demonstrating the potential utility of the intervention in young adults at risk for LTFU. Opportunities for improvement of the intervention included ensuring VL testing and enhanced adherence through closer mentoring and monitoring of case managers to ensure they provide treatment literacy, including education about the benefits of ART, VL testing, and VS (U = U). In addition, closer communication between community and facility case managers could further improve return to care and prevent missed appointments. We demonstrated the limitations of facility-based medical records in capturing retention of highly mobile populations and the potential value of open source POC EMR. Additional evaluations in larger cohorts of KP and PP with longer follow-up time are needed to verify our findings and determine whether long-term retention in care is improved through case management intervention.

## Data Availability

The datasets used and/analysed during the current study are available from the corresponding author upon reasonable request.

## Abbreviations

ART: Antiretroviral therapy
CCM: Community case managers
CD: Cluster of differentiation
CDC: Centers for disease control
EMR: Electronic medical record
FCM: Facility case managers
HIV: Human immunodeficiency virus
KP: Key populations
LTFU: Lost to Follow up
PEPFAR: President’s Emergency Plan for AIDS
POC: Point-of-care
PP: Priority populations
VL: Viral load
VS: Viral suppression
WHO: World Health Organisation
